# Catastrophic ocular complications in leprosy: a case report

**DOI:** 10.11604/pamj.2023.44.198.37051

**Published:** 2023-04-24

**Authors:** Samyak Ashok Ganjre, Sandhya Jeria, Bhushan Madke

**Affiliations:** 1 Department of Dermatology, Venereology and Leprosy, Jawaharlal Nehru Medical College, Datta Meghe Institute of Higher Education and Research, Wardha, Maharashtra, India,; 2 Department of Ophthalmology, Jawaharlal Nehru Medical College, Datta Meghe Institute of Higher Education and Research, Wardha, Maharashtra, India

**Keywords:** Disabilities, deformities, ocular leprosy, staphyloma, case report

## Abstract

Leprosy is a chronic, granulomatous infectious disease commonly affecting the skin, nerves, mucosa and eyes. The stigma associated with the disease frequently leads to delay in presentation to health professionals. Treated patients, though considered cured presumptively, many continue to live with physical disabilities and deformities. Intact visual acuity prevents humans from trauma and any reduction in visual acuity, especially in leprosy, increases the risk of getting injured by many folds. Here, we present a case of leprosy with complete loss of vision due to bilateral anterior staphyloma secondary to keratitis and his physical deformities preventing him to take care of his eyes. This paper aims to emphasize on the importance of a baseline ophthalmology consultation in all newly diagnosed leprosy patients and repeat examination at onset of any new symptoms of the eye.

## Introduction

Leprosy, also called Hansen´s disease, is a chronic infective granulomatous disease caused by *Mycobacterium leprae* [1,2]. With the implementation of MDT (multidrug therapy) in 1981, the rate of cure and prevention of disabilities has certainly reduced globally [3,4]. Ocular involvement in leprosy is 70-75%, about 10-50% of leprosy patients suffer from severe ocular symptoms and blindness occurs in about 5% of patients [5]. Leprosy is considered as a preventable cause of blindness. Leprosy is a common cause of physical disabilities, which leads to social stigma and isolation. Although MDT has reduced the disabilities caused by leprosy, its contribution in reducing the incidence of reactions and subsequent nerve damage has yet to be confirmed. Hitherto, strategies to prevent disabilities in old cured patients and reduce their incidence in the newly diagnosed are still lacking.

## Patient and observation

**Patient information:** a 76-year-old farmer presented with loss of vision in both eyes and amputated digits for the past twenty years. On enquiry, he revealed that he was diagnosed case of leprosy but was irregular with antileprosy drugs and visited multiple healthcare centers for his complaints. Over the past twenty years, he had episodes of redness, pain, watering in both the eyes, for which he had taken treatment from a local practitioner.

**Figure 1:**
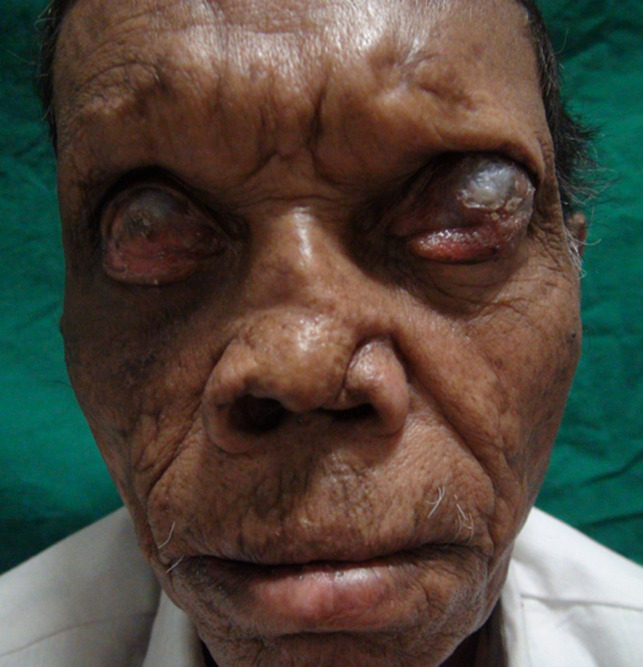
anterior staphyloma in both eyes with madarosis, wide palpebral fissure and collapsed nasal bridge

**Figure 2:**
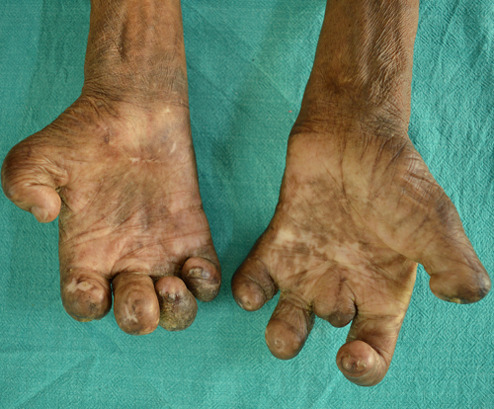
amputated digits of bilateral hands (palmar view)

**Figure 3:**
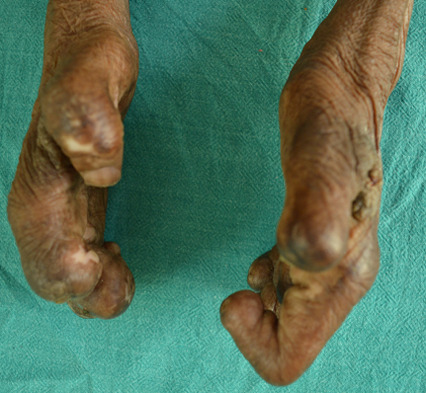
amputated digits of bilateral hands (lateral view)

**Figure 4:**
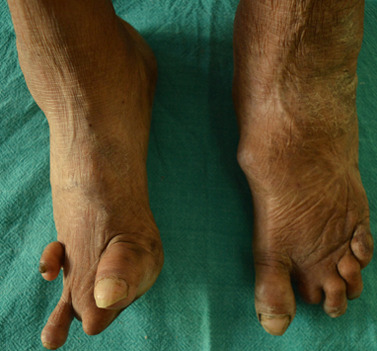
amputated digits of feet

**Clinical findings:** ocular examination of both eyes revealed visual acuity with no perception of light, wide palpebral aperture, complete loss of eyebrows and eyelashes, ectropion of upper and lower lid and extraocular movement restriction in all gazes. There was keratinization of bulbar and palpebral conjunctiva, and anterior staphyloma in bilateral eyes (Figure 1). General examination of the patient revealed collapsed nasal bridge, resorption of multiple fingers (Figure 2, Figure 3), and toes (Figure 4).

**Timeline of current episode:** the patient has been suffering from loss of vision due to leprosy for about twenty years. Over the due course of time, the digits of his hands and toes got amputated.

**Diagnosis:** the patient was a diagnosed case of leprosy.

**Therapeutic interventions:** no therapeutic intervention was provided in the department of dermatology, as the patient was a burnt-out case of leprosy.

**Follow-up and outcome of interventions:** the patient was advised enucleation followed by implantation of orbital implant and orbital prosthesis for cosmetic correction in the department of ophthalmology but got lost to follow up.

**Informed consent:** written informed consent was obtained from the patient.

**Diagnostic assessment:** peripheral nerve examination revealed thickened bilateral ulnar, bilateral median, bilateral radial cutaneous, left lateral popliteal and left anterior tibial nerves. Sensations were absent on the upper and diminished on the lower limbs in a glove and stocking pattern. Slit skin smear (SSS) examination from earlobes and dorsal surface of fingers were negative for acid-fast bacilli.

## Discussion

In Leprosy, eyes can be involved in three ways; (i) as a complication of involvement of facial and trigeminal nerve; (ii) by invasion of the eyeball by *Mycobacterium leprae* in lepromatous leprosy; (iii) and by participation in the generalized allergic reaction, known as the reactive phase [6]. Granulomatous infiltration of branches of facial nerve which supplies the frontalis and orbicularis oculi leads to frontalis weakness and lagophthalmos. Absence of blinking and lagophthalmos predisposes the eye to injuries, foreign bodies and constant exposure of cornea to heat, dust and wind leading to exposure keratitis. Secondary infection of exposure keratitis leads to development of corneal ulcer and perforation. In our patient, loss of corneal sensation and lagophthalmos resulted in development of corneal ulcer, which eventually perforated. Organization of exudates and laying down of fibrous tissue healed the defect, but at the expense of loss of transparency, reduced strength and reduced vision. Gradually the weak anterior surface of the eye-lined by newly formed epithelium protruded outward leading to an anterior staphyloma [7]. A leprosy patient, with intact visual acuity, could manage his daily routine. Leprosy patients with ocular involvement is a double handicap-ocular involvement resulting in loss of visual acuity and blindness, and deformities of limbs resulting in additional disabilities. Appropriate care of affected body part (especially the areas with reduced sensations) is hampered due to poor vision and visual sensory inputs, and deformed extremities in turn do not permit proper eye care. The social stigma, old age of patients, and poor quality of eye care services further add to the woes of the patient. This article presents one such case of leprosy, with resorption of digits and lack of proper eye care leading to bilateral blindness. Other clinical presentations of ocular leprosy are mentioned in Table 1. Inflammation of tissues in the superior orbital fissure (CN III, CN IV and V1 division of CN V and CN VI) and in the optic nerve canal results in Orbital apex syndrome.

**Table 1:** clinical presentations of ocular leprosy

Extraocular		Ocular	
i	Eyebrow: madarosis	i	Conjunctiva: conjunctivitis, conjunctival scarring, pterygium, crystalline clofazamine deposits (in long-term antileprosy MDT)
ii	Eyelid: thickening, entropion, ectropion, nodules on eyelids, ptosis, lagophthalmos, decreased blinking rate	ii	Cornea: corneal nerve beading, corneal hypoesthesia, exposure keratopathy, supericial punctatekeratitis, pannus, infectious keratitis, corneal ulceration, corneal opacification, corneal scarring, keratic precipitates
iii	Eyelash: trichiasis, madarosis	iii	Sclera: scleritis, episcleritis
iv	Nasolacrimal apparatus: dacrocystitis	iv	Iris: iridocyclitis, thinning of iris stroma, miliary iris lepromata (iris pearls), iris atrophy
		v	Uvea: uveitis, uveal infusion, glaucoma
		vi	Pupil: progressive miosis, posterior synechiae
		vii	Lens: cataract
		viii	Ciliary body: low introcular pressure
		ix	Retina: retinal pearls, retinal detachment

## Conclusion

In leprosy, risk of ocular complications increases with the duration of the disease. Visual impairment resulting from leprosy is preventable if diagnosed at early stage. All leprosy patients (including the cured ones) should undergo a baseline ophthalmological examination, and must be made aware that prompt ophthalmological review is required for any new signs or symptoms of the eye to prevent avoidable blindness due to life-long risk of sight-threatening ocular complications. Patients deemed to be at a higher risk warrant regular follow-up.
